# *Staphylococcus aureus* persisters are associated with reduced clearance in a catheter-associated biofilm infection

**DOI:** 10.3389/fcimb.2023.1178526

**Published:** 2023-05-09

**Authors:** Trenten J. Theis, Trevor A. Daubert, Kennedy E. Kluthe, Kenan L. Brodd, Austin S. Nuxoll

**Affiliations:** Department of Biology, University of Nebraska at Kearney, Kearney, NE, United States

**Keywords:** *Staphylococcus aureus*, biofilm, antibiotic tolerance, TCA cycle, persister

## Abstract

**Background:**

*Staphylococcus aureus* causes a wide variety of infections, many of which are chronic or relapsing in nature. Antibiotic therapy is often ineffective against *S. aureus* biofilm-mediated infections. Biofilms are difficult to treat partly due to their tolerance to antibiotics, however the underlying mechanism responsible for this remains unknown. One possible explanation is the presence of persister cells—dormant-like cells that exhibit tolerance to antibiotics. Recent studies have shown a connection between a *fumC* (fumarase C, a gene in the tricarboxylic acid cycle) knockout strain and increased survival to antibiotics, antimicrobial peptides, and in a *Drosophila melanogaster* model.

**Objective:**

It remained unclear whether a *S. aureus* high persister strain would have a survival advantage in the presence of innate and adaptive immunity. To further investigate this, a *fumC* knockout and wild type strains were examined in a murine catheter-associated biofilm model.

**Results:**

Interestingly, mice struggled to clear both *S. aureus* wild type and the *fumC* knockout strains. We reasoned both biofilm-mediated infections predominantly consisted of persister cells. To determine the persister cell population within biofilms, expression of a persister cell marker (P*cap5A::dsRED*) in a biofilm was examined. Cell sorting of biofilms challenged with antibiotics revealed cells with intermediate and high expression of *cap5A* had 5.9-and 4.5-fold higher percent survival compared to cells with low *cap5A* expression. Based on previous findings that persisters are associated with reduced membrane potential, flow cytometry analysis was used to examine the metabolic state of cells within a biofilm. We confirmed cells within biofilms had reduced membrane potential compared to both stationary phase cultures (2.5-fold) and exponential phase cultures (22.4-fold). Supporting these findings, cells within a biofilm still exhibited tolerance to antibiotic challenge following dispersal of the matrix through proteinase K.

**Conclusion:**

Collectively, these data show that biofilms are largely comprised of persister cells, and this may explain why biofilm infections are often chronic and/or relapsing in clinical settings.

## Introduction

*S. aureus* causes a wide variety of infections ranging from minor skin and soft tissue infections to more serious infections such as osteomyelitis, endocarditis, pneumonia, and sepsis (Petti and Fowler, 2002; Ferry et al., 2005; Ziran, 2007). *S. aureus* is the primary mediator for peripheral venous catheter-related infections and one of the leading causes of central venous catheter-related infections with many being recurrent (Arciola et al., 2001; Pujol et al., 2007; Tong et al., 2015). These infections are associated with biofilm formation and can be a prelude to bacteremia (Hoiby et al., 2015). *Staphylococcus aureus* causes an estimated 20-50 cases of bacteremia infection for every 100,000 people annually with a 10-30% mortality rate (van Hal et al., 2012). Interestingly, methicillin susceptible *S. aureus* strains remain difficult to treat in a clinical setting despite susceptibility to antibiotics (Senneville et al., 2011; Conlon, 2014). Biofilm mediated infections are particularly recalcitrant to antibiotic therapy (Costerton et al., 1999; Wenzel, 2007; Olsen, 2015; Svensson Malchau et al., 2021). Several mechanisms have been proposed to explain failure of antibiotic therapy against biofilms, however, one leading hypothesis suggests a subpopulation of persister cells mediates antibiotic tolerance.

Persister cells are a dormant subpopulation of bacteria that exhibits tolerance to antimicrobial agents (Dawson et al., 2011; Balaban et al., 2019). These cells were first discovered by Joseph Bigger, when he observed not all staphylococci cells died following penicillin treatment, and consequently named these cells “persisters” (Bigger, 1944). Since then, persister cells have been documented in a wide variety of pathogenic organisms (LaFleur et al., 2010; Mulcahy et al., 2010; Schumacher et al., 2015; Silva-Herzog et al., 2015; Fisher et al., 2017). While many organisms exhibit persister cells, the mechanism resulting in their formation remained more elusive. *Escherichia coli* persister cell formation has been well documented and originally linked to toxin/antitoxin systems, which leave the cell in a dormant state (Keren et al., 2004; Korch and Hill, 2006; Hansen et al., 2012). Additionally, there is evidence that the stringent response may contribute to antibiotic tolerance in *Pseudomonas aeruginosa* (Nguyen et al., 2011). In *S. aureus*, the mechanism for persister cell formation under antibiotic treatment has been recently uncovered as TCA cycle-, ATP-, and membrane potential-dependent (Conlon et al., 2016; Wang et al., 2018; Zalis et al., 2019; Pandey et al., 2021). Specifically, knockouts of tricarboxylic acid (TCA) cycle genes 2-oxoketoglutarate dehydrogenase (*sucA*), succinyl coenzyme A (CoA) synthetase (*sucC*), and fumarase (*fumC*) exhibited increased multi-drug tolerance (Zalis et al., 2019). Furthermore, disruption of the TCA cycle gene, *fumC*, was found to increase *S. aureus* tolerance to antimicrobial peptides (Hobbs et al., 2021). Given the energy-dependent nature of how these persister cells form, it is not surprising that *S. aureus* persister cells are growth phase dependent. Stationary phase cultures exhibit increased tolerance to antibiotics compared to exponential phase cultures (Mayhall and Apollo, 1980; Eng et al., 1991; Keren et al., 2004; Conlon et al., 2016). Furthermore, high stochastic expression of two stationary phase markers *cap5A* (capsular polysaccharide)and *arcA* (arginine deiminase) has been linked to increased antibiotic tolerance in an exponential phase culture (Conlon et al., 2016). Persisters have been increasingly associated with biofilm tolerance, however the aforementioned mechanisms have not been investigated within a biofilm.

The formation of biofilms is especially problematic in clinical settings due to high antibiotic tolerance and protection from host defense mechanisms (Mah and O'Toole, 2001; Otto, 2018). It was first thought that tolerance of biofilms was due to the protective nature of the matrix (Bolister et al., 1991; Hoyle and Costerton, 1991; Allison and Matthews, 1992; Hoyle et al., 1992; Suci et al., 1994; Kong et al., 2016). However, several studies offer opposing evidence for many antibiotics effectively penetrating biofilms (Anderl et al., 2000; Stone et al., 2002; Zheng and Stewart, 2002; Walters et al., 2003; Rodríguez-Martínez et al., 2007; Singh et al., 2010; Nabb et al., 2019). Therefore, the recalcitrant nature of biofilms is likely due to an alternate mechanism. Specifically, it has been proposed the presence of persister cells mediates antibiotic failure within a biofilm (Lewis, 2007; Conlon et al., 2013). Supporting evidence is provided by biofilms having reduced metabolic activity and high tolerance to antibiotics (Waters et al., 2016), however, further examination is needed to determine the role of persisters within biofilms.

## Materials and methods

### Strains and growth conditions

Methicillin susceptible *S. aureus* strain HG003 was used as a wild type strain in all experiments (Herbert et al., 2010). LACJE2 *fumC::N∑* was obtained from the Nebraska transposon mutant library (transposon insertions designated by: *N∑*, Biodefense and Emerging Infections [BEI] Research Resources Repository, Manassas, VA) and transduced into HG003 using bacteriophage Φ11. Cultures were grown in 3 mL of tryptic soy broth (TSB) in a 14mL snap-cap tube at 225 rpm at 37°C for all experiments and cultures unless otherwise denoted. Overnight cultures were diluted 1:1000 and allowed to grow for another 4 hours to capture the cells in mid-exponential phase unless otherwise denoted.

### Catheter-associated biofilm mouse model

Biofilm mediated infections were established in C57Bl/6 mice following the insertion of a catheter into each flank. Six- to eight-week-old male and female C57Bl/6 were anesthetized using 0.090 mL of ketamine-xylazine per 10 grams of body weight. Flanks of the mice were shaven and two small incisions (~0.25”) were made. Two small catheters (Durect polyethylene catheter tubing #0007750, I.D. 0.030”, O.D. 0.048”; ~0.5” long; one per incision) were inserted and inoculated with 1 x 10^6^ CFU/catheter of either HG003 *fumC::NΣ* or wild type HG003 bacteria. As a control, 10 uL of 1% NaCl was inserted into the catheters of an equal number of mice. Nine days post-infection mice were sacrificed, catheters and surrounding tissues were excised, sonicated or homogenized, serially diluted and subsequently plated on TSA for determination of bacterial burden. For sonication of catheters, each catheter was place in a 1.5 mL microcentrifuge tube with 1 mL 1% NaCl and 15 pulses (500 msec, 50% amplitude) was used to dislodge bacteria from catheters. Sample sizes vary as following due to some mice removing their catheters (Females: Saline (n=8), WT HG003 (n=15), *fumC*::NΣ HG003 (n=8); Males: Saline (n=9), WT HG003 (n=10), *fumC*::NΣ HG003 (n=10)). Statistical significance was determined using a one-way ANOVA with a Tukey test follow-up (p<0.05).

### 96-well static biofilm tolerance assays

Overnight cultures of *S. aureus* were diluted 1:1000 in 200uL TSB within a 96-well flat-bottom plate (Costar #3628) and incubated statically at 37°C for 8 hours (immature biofilm) or 24 hours (mature biofilm). Non-adherent cells were washed with 1% NaCl and biofilms were challenged with 10X MIC of either, ciprofloxacin, rifampicin, gentamycin, oxacillin, and combinations thereof, except for vancomycin which was challenged with 100X MIC. MIC values were previously obtained: vancomycin: 1 µg/ml, ciprofloxacin: 0.5 µg/ml, rifampicin: 0.008 µg/ml, gentamicin: 1 µg/ml, oxacillin: 0.5 µg/ml (Nabb et al., 2019). Following 24 hours of antibiotic treatment, biofilms were washed with 1% NaCl, solubilized, serially diluted, and plated on TSA to enumerate surviving bacteria. Experiments were performed in triplicate and statistical significance was determined using a two-way ANOVA followed by a *post hoc* Sidak’s test (p<0.05).

### Flow cytometry and FACS analysis with *dsRED* reporters

Determination of P*cap5A::dsRED* expression were performed as described previously (Conlon et al., 2016) with the following modifications. Fluorescence of P*cap5A::dsRED* was measured using a Sony SH800 flow cytometer. Populations were determined using forward scatter (FSC) and back scatter (BSC) parameters and fluorescence was detected by emission at 561 nm with a bandpass filter of 600/60 nm. Biofilms were grown for 24 hours as described above, non-adherent cells were washed away with PBS and biofilms were treated with 10X MIC ciprofloxacin and 100X MIC rifampicin in fresh TSB. Following antibiotic challenge for 24 hours, biofilms were washed in PBS, solubilized, and diluted 1:20 in 1 ml of PBS. Events (cells) were gated into the brightest 5%, dimmest 5%, and middle 5-10% based on dsRED fluorescence. Events (cells) were sorted in a 96 well fashion, with 32 spots per gated population. 10 cells per spot were plated for *bright* and *middle* populations, while 100 cells per spot were plated for the *dim* populations to account for the increased killing in this population. To calculate percent survival of each population following antibiotic challenge, untreated biofilms were sorted at one event per spot onto 96 spots (32 dim, 32, middle, 32 bright). Cells were sorted onto rectangular TSA plates and incubated at 37°C for 24 hours before colonies were counted. Percent survival was calculated by taking surviving cells post antibiotic treatment/surviving cells in untreated control * number of events plated per spot. Experiments were performed in replicates of six. Error bars represent standard deviation, and percent survival of each population was compared using a one-way ANOVA and follow-up Tukey tests (p<0.05).

### Measurement of membrane potential in individual cells

The BacLight Bacterial Membrane Potential Kit was used following the manufacturers procedures. Briefly, cultures were grown to either mid-exponential phase, stationary phase, as a 24- or 48- hour biofilm in TSB. Cultures were diluted to 10^6^ CFU/mL in 1 mL PBS. Diethyloxacarbocyanine (DiOC_2_) was used to stain cells for 30 minutes and cultures were subsequently analyzed by flow cytometry (Sony SH800). Cells were excited at 488 nm and the ratio of green to red fluorescence was calculated. As a control, carbonyl cyanide m-chlorophenylhydrazone (CCCP) was added for 5 minutes to dissipate membrane potential. This served as the basis of gating in all graphs. Experiments were performed in triplicate and statistical significance was determined using a one-way ANOVA followed by a Tukey’s test (p<0.05).

### Biofilm dispersal and crystal violet staining assays

HG003 biofilms were grown for 24 hours as described above. Biofilms were dispersed using Proteinase K as described previously with the following modifications (Schaeffer et al., 2016). Twenty-four-hour biofilms were treated with 100 μg/mL proteinase K (Thermo Scientific), in 20 mM Tris-HCl, 100mM NaCl, pH7.5 for 90 minutes at 37°C. Cells incubated in the buffer in the absence of proteinase K served as a control. Following treatment, biofilms were washed twice with PBS. For the crystal violet staining assay, procedures were performed as previously described (Beenken et al., 2003). Briefly, biofilms were stained with 0.4% crystal violet for 2 minutes and excess stain was removed by washing biofilms three times with PBS. Biofilms were solubilized with 200 uL of 100% ethanol and quantified by measuring optical density (OD) at 600nm using a BioTek Synergy H1 microplate reader. To test antibiotic tolerance, 24-hour biofilms were treated with proteinase K as described above; and antibiotic tolerance was determined as described in the *96-Well Static Biofilm Tolerance Assays* methods section. Experiments were performed in triplicate and statistical significance was determined using a two-way ANOVA followed by a *post hoc* Sidak’s test (p<0.05).

### Time dependent kill assay with proteinase K

Overnight cultures were diluted 1:1000 and grown to mid-exponential phase in 3 mL TSB in a 14 mL snap cap tube. Cultures were washed and treated with 100 μg/mL proteinase K (Thermo Scientific), in 20 mM Tris-HCl, 100mM NaCl, pH7.5 for 90 minutes at 37°C at 225 rpm. Cells incubated in the buffer in the absence of proteinase K served as a control. Cultures were washed and then treated with ciprofloxacin (10x MIC) for 24 hours. Samples were serially diluted and then plated on TSB and enumerated. Experiments were performed in triplicate and statistical significance was determined using a two-way ANOVA followed by a *post hoc* Sidak’s test (p<0.05).

### Ethics

All animal work performed in this study was carried out in strict accordance with the guidelines set in the Guide for the Care and Use of Laboratory Animals (National Institutes of Health), the Animal Welfare Act, and the US federal law. Care of the mice was consistent with the American Veterinary Medical Association recommendations. The protocols used in this study were approved by the Institutional Animal Care and Use Committee at the University of Nebraska at Kearney (#200701 and #042717).

## Results

### Catheter-associated biofilm mouse model

**Figure 1:**
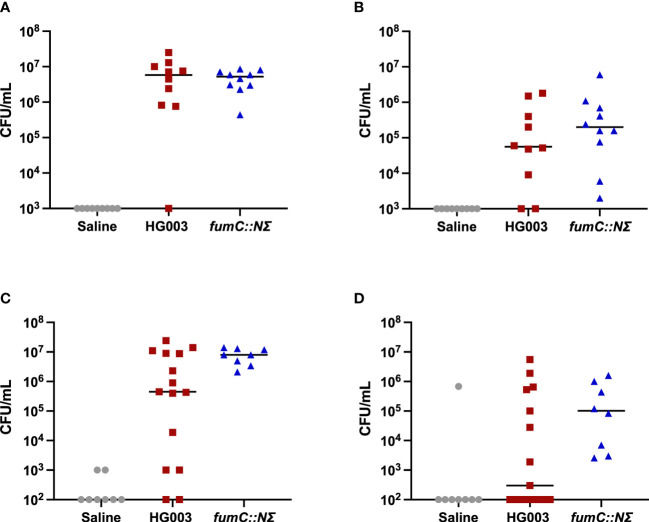
Murine catheter-associated biofilm infection. 6-8 week old male and female C57Bl/6 mice were infected with saline (grey circles), 1x10^6^ CFU/mL HG003 (red squares), or 1x10^6^ CFU/mL HG003 *fumC::NΣ* (blue triangles). Nine days following infection, catheters were removed, sonicated, and plated for surviving bacteria. Tissue surrounding the catheter was homogenized and surviving bacteria were enumerated. Horizontal line represents median log_10_ CFU/mL. Male mice infected with HG003 had similar bacterial burdens as mice infected with *fumC::NΣ* in both **(A)** catheters and **(B)** tissue surrounding the catheter. Female mice infected with HG003 had reduced bacterial burden in the **(C)** catheter (1 log) and **(D)** tissue surrounding the catheter (2.5 logs) compared to female mice infected with *fumC::NΣ.* No statistical significance (>0.05) was shown between populations in either gender using a one-way ANOVA.

The *fumC::N∑* strain in *S. aureus* was previously shown to have increased survival to components of innate immunity (Hobbs et al., 2021). Given this, we sought to determine whether there was increased survival of *fumC::NΣ* in a host with a more robust immune response. C57Bl/6 mice were inoculated in a catheter-associated biofilm model with HG003, *fumC::N∑*, or sterile 1% NaCl. The mice were harvested at 9 days and catheters and surrounding tissue were sonicated and homogenized to determine bacterial burden, respectively (**Figures 1A–D**). Both male and female C57Bl/6 mice were unable to reduce bacterial burden after 9 days of infection from the catheters (1A, 1C). Additionally, male mice failed to reduce bacterial burden in the surrounding tissue (1B) while several female mice infected with wild type *S. aureus* exhibited reduced bacterial burden (1D). While not significantly significant due to the variation in bacterial burden, these findings suggest the *fumC::N∑* strain may have a fitness advantage in a host outside of the catheter biofilm.

### Biofilm-mediated antibiotic tolerance

**Figure 2:**
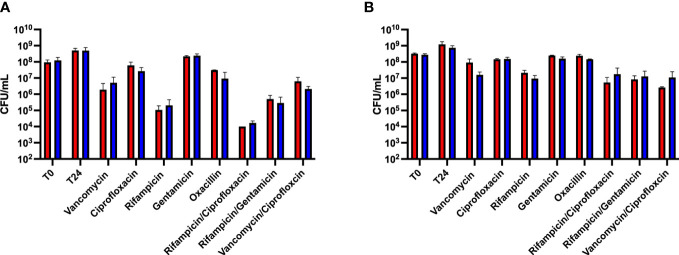
Biofilm-mediated antibiotic tolerance. Overnight cultures of *S. aureus* were diluted 1:1000 in 200 µL TSB in a 96-well microtiter plate. Plates were incubated for 8 hours **(A)** or 24 hours **(B)** at 37°C statically. Non-adherent cells were washed, fresh TSB was added, and biofilms were challenged with antibiotics (10-100x MIC) for 24 hours. Immature biofilms exhibited increased killing compared to mature biofilms; however, no statistical difference was observed between the wild type HG003 (red) and *fumC* knockout (blue) strains. Experiments were performed in triplicate and statistical significance was determined using a two-way ANOVA followed by a *post hoc* Sidak’s test (p<0.05).

To determine whether wild type *S. aureus* exhibited similar levels of antibiotic tolerance as the *fumC::N∑* strain when in a biofilm, a kill assay was performed. Mature biofilms often do not respond to antibiotic treatment, therefore, we sought to determine antibiotic tolerance both within mature biofilms as well as immature biofilms. Antibiotic challenge of HG003 and *fumC::N∑* within immature biofilms showed 0-4 logs of killing, but no significant differences in survival were observed between wild type HG003 and *fumC::NΣ* in any of the eight antibiotic treatment groups (**Figure 2A**). Similarly, antibiotic challenge of HG003 and *fumC::N∑* in mature biofilms resulted in 0-2 logs of killing with no significant differences in bacterial burden observed in any of the antibiotic treatment groups (**Figure 2B**). These experiments demonstrate that wild type HG003 biofilms have similar antibiotic tolerance compared to *fumC::NΣ* HG003 biofilms, and that mature biofilms have increased tolerance compared to immature biofilms.

### Persister cell analysis

**Figure 3:**
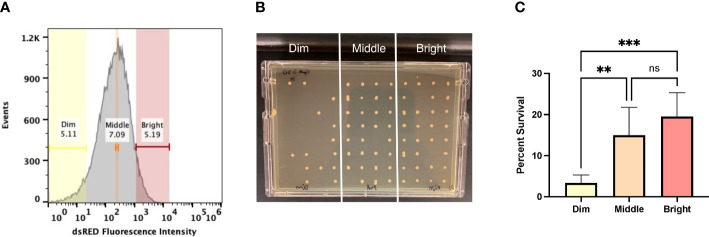
Persister marker analysis. **(A)** 24-hour biofilms were exposed to ciprofloxacin (10x MIC) for 24 hours and expression of *cap5A* was measured using FACS. The antibiotic-challenged biofilms were gated into three populations based on dsRED fluorescence. The lowest dsRED expressing cells (dim, yellow), cells with medium levels of fluorescence (middle, orange), and those with the highest levels of dsRED fluorescence (bright, red). **(B)** Cells were sorted at 10 events/spot and incubated for 24 hours at 37°C, statically. **(C)** The bright and middle fractions were statically more likely to survive antibiotic challenge. Experiments were performed in replicates of six and statistical significance was determined using a one-way ANOVA followed by a *post hoc* Tukey’s test (**p<0.005, ***p<.0005).

One mechanism that could explain the similar levels of antibiotic tolerance between HG003 and *fumC::N∑* within a biofilm as well as in the *in vivo* catheter biofilms is similar levels of persister cell formation between the two strains. To determine whether this was the case, a persister cell marker was used to examine whether survival in a biofilm following antibiotic treatment was associated with persister formation. A capsular polysaccharide biosynthesis gene (*cap5A*) was previously demonstrated to be a persister marker in exponentially growing cells following ciprofloxacin treatment (Conlon et al., 2016). *S. aureus* HG003 P*cap5A::dsRED* was grown for 24 hours in a biofilm state and analyzed by fluorescence-activated cell sorting (FACS) following a lethal dose of ciprofloxacin (10x MIC). Cells within the biofilm were gated based on expression of P*cap5A::dsRED* into “bright”, “middle”, and “dim” populations onto a TSA plate to enumerate surviving cells (**Figures 3A, B**). Compared to the dim population, the middle and bright populations had 4.5x and 5.8x more survivors, respectively (**Figure 3C**).

**Figure 4:**
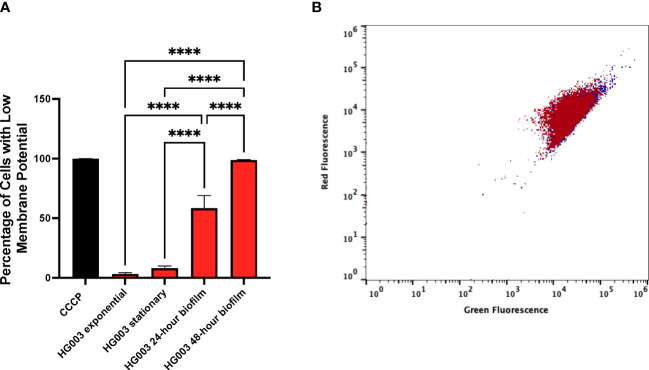
Membrane potential within biofilms. **(A)** Membrane potential was measured from either wild type *S. aureus* or *fumC::NΣ* in exponential phase, stationary phase, and within 24- and 48-hour biofilms. 1x10^6^ cells in PBS were stained with DiOC_2_(3) for 30 minutes prior to flow cytometry analysis. Carbonyl cyanide *m-*chlorophenylhydrazone (CCCP) was used as a control for gating low membrane potential cells. A small fraction of cells from exponential cultures exhibits reduced membrane potential compared to those grown in stationary phase. Cells grown in a biofilm have a further increase in the percentage of cells with low membrane potential, **(B)** however no difference was observed between wild type (red) and *fumC::N∑* (blue). Experiments were performed in triplicate and statistical significance was determined using a one-way ANOVA followed by a Tukey’s test (****p<0.0001).

In addition to using a persister marker to characterize biofilms, membrane potential of the cells within a biofilm was examined. Previous work demonstrated *S. aureus* persisters in planktonic cultures are associated with reduced membrane potential (Wang et al., 2018). Membrane potential was measured from exponentially growing cells, stationary phase cells, and from cells growing within a biofilm (**Figure 4A**). Carbonyl cyanide m-chlorophenylhydrazone (CCCP) dissipates membrane potential and was used as a control for gating low membrane potential cells. Only a small fraction of exponentially growing cells (3.1%) exhibited low membrane potential fitting with the observation that most of these cells are not persister cells. Unexpectedly, a large difference in membrane potential between stationary and biofilm cells was observed suggesting cells within a biofilm are less metabolically active than stationary phase cells. In line with HG003 and *fumC::N∑* biofilms responding similarly to antibiotics, there was no difference in membrane potential between these strains (**Figure 4B**). Collectively, these results suggest wild type biofilms largely consist of persisters and a *fumC* knockout does not have increased persister formation within a biofilm as was previously observed in planktonic cultures.

### Biofilm dispersal

**Figure 5:**
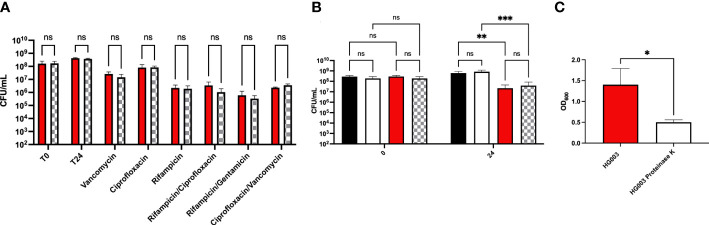
Antibiotic tolerance in dispersed biofilms. **(A)** Overnight *S. aureus* cultures were diluted 1:1000 in 200 µL TSB in a 96-well microtiter plate. Plates were incubated for 24 hours at 37°C, statically. Biofilms were washed with PBS and incubated at 37°C, statically, in either 100 µg/mL proteinase K in proteinase K buffer (20 mM HCl Tris, 100mM NaCl, pH 7.5) (checkered bars) or buffer alone (red bars). Non-adherent cells were washed, fresh TSB was added, and biofilms were challenged with antibiotics (10-100x MIC) for 24 hours. There was no statistical difference in survival to antibiotic challenge following dispersal of biofilms indicating that biofilm mass is not required to confer tolerance. **(B)** Time dependent kill assays were performed on exponential phase cultures in the presence or absence of proteinase K for 24 hours to determine whether the enzyme induced antibiotic tolerance. In the absence of ciprofloxacin, no difference was observed in growth between cells incubated in the presence of proteinase K (white bars) compared to those incubated in buffer alone (black bars). Similarly, following antibiotic treatment, no difference in survival was observed between cultures incubated with proteinase K (checkered bars) compared to buffer alone (red bars). **(C)** Crystal violet staining was performed to confirm biofilm dispersal following proteinase K treatment. Experiments were performed in triplicate and statistical significance was determined using a two-way ANOVA followed by a *post hoc* Sidak’s test (*p<0.05, **p<0.01, ***p<0.001) or using a student’s t-test for the crystal violet staining.

To rule out the possibility of biofilm matrix inhibiting antibiotic activity, antibiotic activity was assessed against biofilms as well as dispersed biofilms. Mature biofilms were treated with proteinase K or buffer alone as a control followed by washing and the addition of lethal concentrations of antibiotics. No differences were observed in bacterial survival following dispersal of biofilm matrix indicating the biofilm matrix is not impacting antibiotic tolerance (**Figure 5A**). To ensure proteinase K treatment didn’t induce antibiotic tolerance, HG003 was grown to exponential phase, treated with proteinase K, washed, and challenged with a lethal concentration of antibiotic for 24 hours. No difference was observed between the proteinase K treated and untreated groups (**Figure 5B**) despite significant reduction in biofilm matrix as indicated by crystal violet staining (**Figure 5C**). These results support the notion that the metabolic state of bacteria within the biofilm confers antibiotic tolerance and not the matrix itself.

## Discussion

In this study, persister cells were explored as mechanism for antibiotic tolerance within a biofilm and as a mechanism for survival within a murine host. It was found that mice were unable to clear *S. aureus* during a catheter-associated biofilm infection. Biofilms were examined for persister cell composition as a mechanism for the increased persistence of *S. aureus* during the murine biofilm infection. *S. aureus* biofilms had multiple attributes associated with persister cells including reduced membrane potential and high expression of a persister cell marker, *cap5A.* Additionally, cells with higher expression of *cap5A* were more likely to survive lethal antibiotic challenge compared to cells with low *cap5A* expression. The matrix of biofilms inhibiting antibiotic diffusion was not observed in this study. Overall, these studies highlight the importance of persister cells within biofilms and survival within a host.

Biofilms are estimated to be associated with upwards of 80% of all microbial infections and are particularly recalcitrant to antibiotic therapy (Davies, 2003; Wolcott et al., 2010; Archer et al., 2011; Joo and Otto, 2012; Jamal et al., 2018). This phenomenon isn’t unique to infections caused by drug-resistant pathogens as biofilm-mediated infections caused by drug-susceptible pathogens are often just as difficult to completely eradicate (Monack et al., 2004; Lewis, 2010; Conlon, 2014). Recent reviews hypothesize antibiotics are ineffective against biofilms due to the presence of persister cells (Lewis, 2010; Conlon, 2014). However, studies investigating the presence of persister cells within a biofilm are largely lacking. Our data provide an underlying mechanism for biofilm tolerance with the presence of persister cells and reduced metabolic activity in the vast majority of cells within a biofilm.

Decreased tricarboxylic acid cycle activity was recently implicated in *S. aureus* antibiotic and antimicrobial peptide tolerance (Zalis et al., 2019; Hobbs et al., 2021). Furthermore, a TCA cycle knockout exhibited increased persistence within a *Drosophila melanogaster* sepsis infection model (Hobbs et al., 2021). We reasoned disruption of the TCA cycle would also provide a fitness advantage in a murine model of infection. Previous work in *S. aureus* demonstrated robust biofilms formed by day 7 in a catheter model and rifampicin and daptomycin treatment was ineffective in reducing bacterial burden (Hanke et al., 2013). In contrast to these findings, treatment of deep-seated mouse thigh infections with vancomycin reduced bacterial burden by nearly 2 logs (Conlon et al., 2013). While these studies use different strains, these data highlight the challenge posed by biofilm mediated infections. To determine if the *fumC::N∑* strain was better able to evade host immunity and persist at a higher bacterial burden, we examined bacterial survival in a catheter-associated biofilm infection. Unexpectedly, mice infected with either HG003 or *fumC::NΣ* had similar difficulty reducing bacterial burden in the catheters. Female mice infected with HG003 were more effective in reducing bacterial burden from the tissue surrounding the catheter compared to female mice infected with *fumC::NΣ* or male mice infected with either strain. The high bacterial burden in the catheters is likely due to biofilm formation on the foreign device, which is in accordance with previous work (Thurlow et al., 2011; Hanke and Kielian, 2012; Hanke et al., 2013). Interestingly, female mice effectively cleared HG003 infections from the surrounding tissue in 46.67% of the infections compared to 0% of infections mediated by *fumC::N∑.* These results suggest outside of the biofilm matrix, such as the tissue surrounding the catheter, increased persister formation may provide a fitness advantage.

Multiple reviews have speculated that biofilms are difficult to eradicate due to the presence of persisters but studies investigating the persister composition within biofilms are largely lacking. Indeed, if persisters predominantly constitute biofilms, this would support the findings in catheter-associated biofilm infections. Previous work characterized the capsular polysaccharide type 5 biosynthesis gene, *cap5A*, as a persister marker in planktonic cultures (Conlon et al., 2016). Cells within a biofilm that had middle to bright expression of P*cap5A::dsRED* were statistically more likely to survive antibiotic treatment. This is slightly contrasting with previous work where only the cells with the highest expression of *cap5A* were more likely to survive antibiotic treatment (Conlon et al., 2016). The primary difference is the previous work examined persisters within a planktonic exponential phase culture compared to biofilms in this study. This also allowed us to speculate that biofilms are composed of a higher percentage of persister cells.

Further support that biofilms are primarily composed of persister cells is provided by experiments examining membrane potential of cells within a biofilm. Recently, it was demonstrated that persister cells have low membrane potential compared to non-persister cells (Wang et al., 2018). Given that the mechanism for persister formation in *S. aureus* is associated with decreased intracellular ATP concentrations and decreased TCA cycle activity, it is fitting that persister cells have lower membrane potential. In agreement with the FACS biofilm data, cells within biofilms had reduced membrane potential compared to stationary and exponential phase planktonic cultures. While a number of studies have shown the majority of antibiotics are not physically impaired by biofilm matrix, biofilm dispersal assays were conducted to offer further support of antibiotic tolerance being mediated through the metabolic state of bacteria within a biofilm. It could be assumed that if the biofilm matrix was contributing to the high degree of antibiotic tolerance, there would be a reduction in bacterial burden following biofilm matrix dispersal. However, the high degree of antibiotic tolerance was consistent following dispersal of biofilm matrix across all classes of antibiotics used. These data are suggestive of a high percentage of persister cells within a biofilm.

Interestingly, and in contradiction to our results, increased *fumC* expression has been associated with *S. aureus* small colony variants (SCVs) (Wong Fok Lung et al., 2020). SCVs are often characterized as slowly growing variants due to mutations in either menadione or heme biosynthesis genes and are frequently associated with chronic infections (Proctor et al., 1995; Proctor et al., 2006; Kahl et al., 2016; Wolter et al., 2019). As a result of these mutations, SCVs frequently are associated with reduced membrane potential and intracellular ATP concentrations leading to aminoglycoside and antimicrobial peptide resistance (Balwit et al., 1994; Samuelsen et al., 2005; Proctor, 2019; Tuchscherr et al., 2020). Persisters and SCVs share reduced membrane potential, however, the increased expression of *fumC* observed in SCVs differs from what has been observed in *S. aureus* persisters. Interestingly, despite these differences in *fumC* expression, both phenotypes are associated with chronic infections in *S. aureus.* A likely explanation is the decrease in membrane potential and ATP concentrations confers tolerance to antibiotics and host immunity despite high *fumC* expression in SCVs. Alternatively, it was recently found fumarase prevents activation of trained immunity through depletion of fumarate (Arts et al., 2016; Prince and Wong Fok Lung, 2020); another plausible explanation is that both phenotypes contribute to persistence within a host through independent mechanisms.

Biofilms are notoriously difficult to eradicate, regardless of the resistance profile of the organism causing the infections. Mounting evidence suggests it is the metabolic state of the cells within the biofilm and not physical inhibition of antibiotics through the matrix of the biofilm (Anderl et al., 2000; Stone et al., 2002; Zheng and Stewart, 2002; Walters et al., 2003; Rodríguez-Martínez et al., 2007; Singh et al., 2010; Conlon, 2014; Waters et al., 2016; Nabb et al., 2019). Reduced membrane potential within biofilms and surviving cells exhibiting increased expression of the persister marker offer further support of this notion. Additionally, these results provide an underlying mechanism for the high degree of antibiotic tolerance in biofilms through persister cells. Recent studies have demonstrated persister cells have increased to survival to components of the host immune system (Stapels et al., 2018; Hobbs et al., 2021). Consistent with those studies, the failure of mice to reduce bacterial burden in the catheter biofilms as well as the female mice being less effective in clearing the *fumC::N∑* strain suggest persisters provide a fitness advantage during infection. These recent studies involving persisters interacting with the immune system may offer an explanation for the chronic and relapsing nature of biofilm-mediated infections. Understanding this may hold broader implications for improving treatment of biofilm infections in a clinical setting.

## Data availability statement

The original contributions presented in the study are included in the article/supplementary material. Further inquiries can be directed to the corresponding author.

## Ethics statement

The protocols used in this study were approved by the Institutional Animal Care and Use Committee at the University of Nebraska at Kearney (#200701 and #042717).

## Author contributions

TT, TD, KK, and AN contributed to the conception and design of the study. TT and AN performed the statistical analysis. TT wrote the first draft of the manuscript. TT and AN wrote sections of the manuscript. TT, TD, KK, KB, and AN performed the experiments, generated data appearing in the manuscript, contributed to the revision of the manuscript, and read and approved the submitted version.
